# Pulmonary effects of inhalation of spark-generated silver nanoparticles in Brown-Norway and Sprague–Dawley rats

**DOI:** 10.1186/s12931-016-0407-7

**Published:** 2016-07-19

**Authors:** Joanna Seiffert, Alison Buckley, Bey Leo, Nicholas G. Martin, Jie Zhu, Ranran Dai, Farhana Hussain, Chang Guo, James Warren, Alan Hodgson, Jicheng Gong, Mary P. Ryan, Junfeng (Jim) Zhang, Alexandra Porter, Terry D. Tetley, Andrew Gow, Rachel Smith, Kian Fan Chung

**Affiliations:** Airways Disease, National Heart & Lung Institute, Imperial College London, Dovehouse St, London, SW3 6LY, UK; Nanoparticle Inhalation Research Group, Public Health England, Oxfordshire, UK; Department of Material Science, Chemistry and the London Centre for Nanotechnology, Imperial College, London, SW3, UK; Department of Clinical Biochemistry, Imperial College Healthcare NHS Trust, Charing Cross Hospital, London, W6 8RF, UK; Nicholas School of Environment & Duke Global Health Institute, Duke University, Durham, USA; Department of Pharmacology and Toxicology, Rutgers University, Piscataway, NJ USA

**Keywords:** Lungs, Silver nanospheres, Inhalation, Inflammation

## Abstract

**Background:**

The increasing use of silver nanoparticles (AgNPs) in consumer products is concerning. We examined the potential toxic effects when inhaled in Brown-Norway (BN) rats with a pre-inflammatory state compared to Sprague–Dawley (SD) rats.

**Methods:**

We determined the effect of AgNPs generated from a spark generator (mass concentration: 600–800 μg/mm^3^; mean diameter: 13–16 nm; total lung doses: 8 [Low] and 26–28 [High] μg) inhaled by the nasal route in both rat strains. Rats were sacrificed at day 1 and day 7 after exposure and measurement of lung function.

**Results:**

In both strains, there was an increase in neutrophils in bronchoalveolar lavage (BAL) fluid at 24 h at the high dose, with concomitant eosinophilia in BN rats. While BAL inflammatory cells were mostly normalised by Day 7, lung inflammation scores remained increased although not the tissue eosinophil scores. Total protein levels were elevated at both lung doses in both strains. There was an increase in BAL IL-1β, KC, IL-17, CCL2 and CCL3 levels in both strains at Day 1, mostly at high dose. Phospholipid levels were increased at the high dose in SD rats at Day 1 and 7, while in BN rats, this was only seen at Day 1; surfactant protein D levels decreased at day 7 at the high dose in SD rats, but was increased at Day 1 at the low dose in BN rats. There was a transient increase in central airway resistance and in tissue elastance in BN rats at Day 1 but not in SD rats. Positive silver-staining was seen particularly in lung tissue macrophages in a dose and time-dependent response in both strains, maximal by day 7. Lung silver levels were relatively higher in BN rat and present at day 7 in both strains.

**Conclusions:**

Presence of cellular inflammation and increasing silver-positive macrophages in lungs at day 7, associated with significant levels of lung silver indicate that lung toxicity is persistent even with the absence of airway luminal inflammation at that time-point. The higher levels and persistence of lung silver in BN rats may be due to the pre-existing inflammatory state of the lungs.

## Background

Silver nanoparticles (AgNPs) are often suspended in a liquid which can be aerosolised or used in a solid form and are widely used as anti-microbials in consumer products especially for textiles, personal hygiene products, medical equipment, wound dressings, respiratory devices, catheters and disinfectant sprays. Therefore, humans run the risk of inhaling AgNPs [1]. What is known about the potential pulmonary toxicity of inhaled AgNPs has been limited to studies in rodents. Subchronic inhalation of AgNPs induced mild, dose-dependent pulmonary inflammation and alterations in pulmonary function [2–6], with evidence that inhaled AgNPs may also enter the systemic circulation to become distributed to extra-pulmonary organs such as the liver and brain [2, 7, 8]. Exposure of rats to AgNP aerosols for 90 days led to modest increased pulmonary function changes, with evidence of genotoxicity and accumulation of tissue macrophages [9], with persistence of lung function and inflammatory changes for up to 12 weeks after cessation of exposure [10]. On the other hand, studies using lower inhaled doses have reported minimal or no toxicity [7, 11–13]. By contrast, direct instillation of AgNPs produced higher levels of inflammation, oxidative stress and cytotoxicity compared to AgNP inhaled at similar doses [14–16].

In our previous study, the inflammatory response induced by intratracheal instillation of AgNPs was dependent on particle size with a greater pulmonary inflammatory response with a 20 nm size than an 110 nm size but with little influence imposed by citrate or polyvinyl phosphate-capping [15]. We also observed a pulmonary inflammatory response characterised by an intense eosinophilia and neutrophilia in the Brown-Norway (BN) rat compared to a predominantly neutrophilic response in Sprague–Dawley (SD) rats. The response to AgNPs by direct inhalation into lungs, which would be the main route of exposure if humans were exposed to AgNPs from consumer products, remains unclear particularly in the potential differences in the response between the 2 rat strains. The BN rat is known to readily develop features of allergic asthma, namely lung eosinophilia and bronchial hyperresponsiveness (BHR) following sensitisation and exposure to allergens and possess features of chronic lung inflammation in contrast to the SD rat [17–20].

AgNPs from consumer products are more likely to be inhaled, and the potential toxic effects of AgNPs will certainly depend on the route on entry of these NPs. Therefore, in order to characterise further the bioreactivity of inhaled AgNPs in the lungs, we have studied the effect of freshly-generated uncoated silver nanoparticles produced by a spark discharge generator. We determined the threshold lung deposited dose on the lung inflammatory response and cytokine levels. An important determining factor of the pulmonary response is the interactions of these particles with the lung lining fluid constituents that include dipalmitoylated phospholipid (DPPC) and surfactant-specific proteins A, B, C and D, which influence the aggregation, dissolution and uptake of these nanoparticles by pulmonary macrophages and alveolar cells and hence their cytotoxicity [21–23]. We therefore measured the distribution of the silver particles in the lungs, assayed silver levels, and related these to the surfactant composition and lung function changes. We determined whether there would be differences in these parameters between the 2 rat strains.

## Methods

### Generation of silver nanoparticle aerosol and exposure system

Aerosols of AgNPs were generated using a spark generator (DNP 4000, Palas, Karlsruhe, Germany) by the homogeneous nucleation of vapour produced between two electrodes (5 mm length and 1 mm diameter silver wire; Premion™ 99.999 % purity, Alfa Aesar™, Heysham, UK) in an inert argon atmosphere at a flow rate of 5 L min^−1^, as previously described [24]. The rate of primary particle production and final size was dependent on the sparking frequency (90–300 Hz). The particles were passed through a krypon-85 charge neutraliser (Model 3077A, TSI Incorporated, Shoreview, MN, USA) and were diluted with oxygen and nitrogen to give a total aerosol flow-rate of 9 L min^−1^. This was led into a custom-built nose-only exposure manifold consisting of 4 chambers as previously described [24]. Animals were held in restraining tubes attached to the chamber ports, thus each had an individual aerosol supply directed to the nose area.

#### Characterisation and monitoring of AgNPs

Aerosol mass concentrations were determined gravimetrically using Pallflex® emfab™ filters (Pall Life Sciences, Ann Arbor, MI, USA) with the aerosol drawn at 2 L min^−1^ with continuous monitoring using a TEOM™ ambient particulate monitor (Model 1400a, Thermo Scientific, Franklin, MA, USA) [24]. The AgNP aerosol was immediately diluted to prevent coagulation using a Palas® ejector dilution system (Model VKL 100, Palas GmbH, Karlsruhe, Germany). HEPA-filtered compressed air was supplied to the diluter, drawing a sample flow rate of 0.12 L min^−1^ from the exposure chamber and giving a dilution ratio of 150 ± 3 %. A condensation particle counter (CPC model 3775, TSI Inc., Shoreview, MN, USA) continuously monitored the concentration of particle numbers with aerosol particle size distribution determined using a scanning mobility particle sizer (SMPS; model 3936 N76, TSI Inc., Shoreview, MN, USA) and aerosol particle shape using high resolution transmission electron microscopy (TEM) (JEOL 3000 F, JEOL Inc., Tokyo, Japan) [24]. Projected area equivalent diameters were calculated for >1500 particles randomly selected using the image analysis software Image J (http://imagej.nih.gov/ij/).

#### Dose estimation

Estimates of the deposited dose, D (μg), in the lung and alveolar regions were determined using the formula, D = C × MV × T × DE × 10^−6^, where C (μ/m^3^) is the aerosol mass concentration, MV (ml/min) the rat minute ventilation, T (min) the exposure duration, and DE the deposition efficiency. The minute ventilation was derived from measurements of tidal volume (TV) and breathing frequency (f) of 6 rats of similar weight to those used in this study made using head-out plethysmograph (EMMS, Bordon, UK) for the duration of nose-only inhalation experiments similar to those described here. The average MV, TV and f were 190 mL/min, 1.6 ml and 130 min^−1^ respectively. The deposition efficiencies for the lung and alveolar regions, of 31 and 21 %, respectively, were calculated using the multiple-path particle dosimetry (MPPD) model (version 2.11, Applied Research Associates, Inc.) [25] using the aerosol CMD and GSD (Table 1) and the breathing parameters indicated above.

**Table 1:** Characterisation of spark generated silver nanoparticles and lung burden on each of the exposure conditions for the two rat strains

	Sprague–Dawley rats			Brown-Norway rats		
	Control	Low dose	High dose	Control	Low dose	High dose
Count Median Diameter (nm)	-	13.4 ± 1.0	14.1 ± 2.3	-	15.9 ± 0.8	15.7 ± 2.8
Geometric Standard Deviation	-	1.60 ± 0.03	1.58 ± 0.06	-	1.58 ± 0.02	1.56 ± 0.06
Concentration /cm^3^	<1	(4.50 ± 0.21) x 10^7^	(4.55 ± 0.70) x 10^7^	<1	(3.89 ± 0.18) x 10^7^	(3.68 ± 0.48) x 10^7^
Mass concentration (μg/m^3^)	-	801 ± 33	670 ± 49	-	791 ± 32	617 ± 25
Exposure duration (mins)	720	180	720	720	180	720
Lung burden (μg)	0	8	28	0	8	26
Alveolar dose (μg)	0	6	19	0	6	18

#### Study design

The experiments were performed within the legal framework of the United Kingdom under a Project License granted by the Home Office of Her Majesty’s government. The researchers hold Personal Licenses provided by the Home Office to perform the experiments in the rat species described here (Project Licence number: PPL 70/7581). These experiments were approved by the Imperial College BioSciences Animal Ethics Committee.

Male pathogen-free Sprague–Dawley (SD) (10–12 weeks, 250–320 g) and Brown-Norway (BN) rats (10–12 weeks, 260–380 g) were purchased from Harlan, UK and housed under filter tops. Rats were randomly assigned into groups and exposed for 3 h on one day (low dose) or for 3 h on four consecutive days (high dose). Three exposure conditions were defined: filtered air only controls for 3 h on 4 consecutive days, low dose AgNP exposure and high dose AgNP exposure (Table 1). Following exposure, the rats were returned to their cages for either 24 h or 7 days.

#### Respiratory mechanics

Respiratory mechanics was measured by the forced oscillation technique at pulmonary end- expiratory pressures (PEEPs) of 3 cm H_2_O. Rats were anesthetised with ketamine (80 mg/Kg) and Xylazine (10 mg/Kg) i.p. and the depth of anaesthesia was quantified by loss of pedal reflex and by pulse oximetry, using the MouseOx Plus [STARR Life Sciences Corp., Oakmont, PA]. Rats were ventilated through a tracheostomy (10 mL/Kg air at 90 breaths per minute) using a computer-controlled ventilator (Spira, EMMS, UK). Three successive deep lung inflations (30 cmH_2_0) were performed to standardise volume history. Tdal breathing was interrupted by an 8 s broadband input signal, containing multiple frequencies between 0.5 and 20Hz. Respiratory impedance was calculated at each frequency using the Fast Fourier transformation of the pressure and flow signals. As a function of frequency, the impedance (Zrs) data can be separated into both resistance (R_L_) and elastance (E_L_) spectrawhich were fitted to a constant phase model which partitions the respiratory mechanics into the central airway (Rn) and coefficients of tissue damping (G) and tissue elastance (H) [26].

#### Bronchoalveolar lavage (BAL)

BAL was performed as previously described [15]. Differential cell counts were performed on cytology of BAL. BAL supernatants were stored for analysis of various analytes.

#### Tissue processing and staining

Paraffin blocks were prepared from lungs and sections stained with hematoxylin and eosin (H & E) and carbol-chromotrope for visualisation of eosinophils as previously described [15]. The lung inflammatory response was measured on a 0–3 scale, as previously defined [15]. Eosinophils in the lamina propria of largest airways in each lung section were counted around 5 × 2nd-3rd generation large airways of ~10 mm in length. Eosinophils up to 2 mm from each airway were counted and expressed as eosinophils per millimetre of basement membrane length.

We also used a Silver Enhancing Kit (Cat no: SE100, Sigma-Aldrich, Saint Louis, USA) to visualise silver nanoparticles using light microscope with positive cells appearing black. Uptake into cells in walls of bronchi or blood vessels, alveoli septa and alveolar space of the left lung lobe were counted. Twenty fields covering the whole left section were counted and data are expressed as number of positive silver cells per field.

#### Measurement of silver in lungs

We used different inductively-coupled plasma mass spectrometry (ICP-MS) methods for each rat species. For the Sprague–Dawley rats, the concentration of silver in lung lobes was quantified by 7700 ICPMS machine (Agilent Technologies) following digestion of tissue samples. A standard reference material was analysed with samples (National Research Council Canada (LUTS-1) non-defatted lobster hepatopancreas) and the detection limit for the analysis was 5 ng per sample. For the Brown-Norway rats, we used 7900 ICPMS machine (Agilent Technologies) on snap-frozen tissue. After freeze drying, tissues were digested by microwave and ICPMS performed in no-gas mode using mass 107 as the quantifier and mass 71 as the internal standard. The ICP-MS was calibrated using silver standards diluted from a 1 ppm stock.

#### Measurement of malondialdehyde

BAL malondialdehyde (MDA) was measured using a HPLC system with fluorescent detection (Waters, Milford, MA, USA) set at 532 nm for the excitation wavelength and 553 nm for the emission wavelength. A Nova-Pak C18 column (Waters, Milford, MA, USA) was used with a mobile phase that was composed of 40 methanol and 60 % water containing 50 mM KH_2_PO4 (pH = 6.8). The detection limit, extraction recovery and analytical precision were 1.8 nM, 75.9, and 2.2 %, respectively.

#### Total phospholipid and surfactant proteins in BAL

BAL supernatant was separated into two fractions using differential centrifugation. 1 mL of BAL from the first wash was centrifuged at 18,000 rpm for 30 min at 4 °C to obtain large and small aggregate fractions. The large aggregate (LA) fraction (pellet) contains phospholipids, tubular myelin, lamellar bodies, large vesicles, SP-A, B and C. The supernatant contains the small aggregate fraction (SA) consisting of small vesicles, SP-D and little surface functional surfactant [27]. The LA was re-suspended in 40 μL of physiological saline and 5 μL was separated into organic and aqueous fractions by a chloroform and methanol extraction. The lower layer containing the organic fraction was dried under nitrogen. Total organic phosphorus was extracted using a perchloric acid digestion (70 %) for one hour at 200 °C, with potassium phosphate standards treated in the same way as the samples and assayed using the method of Bartlett [28]. Organic phospholipid was expressed as μg of total organic phosphate in 5 μL LA, equivalent to 1/5 mL of BAL. Protein concentrations in the LA and SA were determined by the Bio-rad assay. SP-D was measured in whole BAL supernatants by ELISA [Cusabio Biotech Co., Suffolk, UK] and SP-B inthe LA fraction by ELISA [Cusabio Biotech Co., Newmarket, Suffolk, UK.

#### Cytokine and chemokine levels in BAL

Cytokines and chemokines including KC, CCL11 (eotaxin), Interferon (IFN)-γ, IL-1β, IL-4, IL-6, IL-13, IL-17A, CCL2 (MCP-1) and CCL3 (MIP-1α) were measured in BAL supernatants using a Milliplex MAP rat cytokine panel (Millipore Analayte Kit Finder, Millipore Ltd, Watford, UK) according to the manufacturer’s specifications.

#### Data analysis

Data analysis was performed using Prism 5 software. Data were treated non-parametrically as data was generally not normally distributed when tested using the Shapiro-Wilk normality test. A non-parametric ANOVA (Kruskal-Wallis test) was performed at each time point and comparison of the means of the multiple groups was assessed by Dunn’s post-hoc test. P values <0.05 were considered significant.

## Results

### AgNP dose in the lungs

**Fig. 1:**
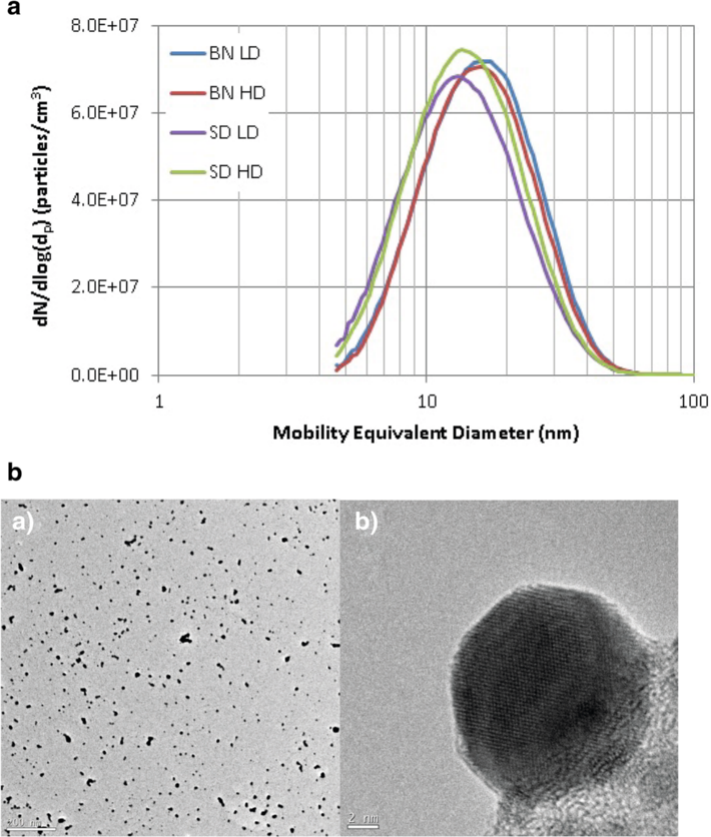
Panel **a**: Density of particles as a function of the diameter of silver nanoparticles measured during each of the experimental conditions for each rat strain. BN-LD: Brown Norway rats exposed to low dose; BN-HD: Brown Norway rats exposed to high dose; SD-LD: Sprague–Dawley rats exposed to low dose; SD-HD: Sprague–Dawley rats exposed to high-dose. Panel **b**: Representative high resolution transmission electron microscopy (TEM) images of aerosol particles delivered to the exposure manifold at 12,000X magnification (scale bar 200 nm) (Panel **B** a) and 800,000X magnification (scale bar 2 nm) (Panel **B** b)

The AgNPs had an average count median diameter (CMD) spanning between 13.4 ± 1.0 nm and 15.9 ± 0.8 nm on the different days of exposure with particle number concentrations between 3.68 ± 0.48 × 10^7^ and 4.55 ± 0.70 × 10^7^/cm^3^ (Table 1; Fig. 1). Mass concentrations measured gravimetrically ranged from 617 ± 25 to 801 ± 33 μg/m^3^. Figure 1 shows representative images of separate Ag nanospheres collected during exposures, illustrating their spherical form. In SD rats, low and high lung doses were estimated using deposition fractions from the MPPD model as 8 and 28 μg respectively, while in BN rats, these were 8 and 26 μg. The equivalent values for alveolar deposition were 6 and 19 μg for the SD rats and 6 and 18 μg for the BN rat.

#### Quantification and localisation of silver in lung tissue

**Fig. 2:**
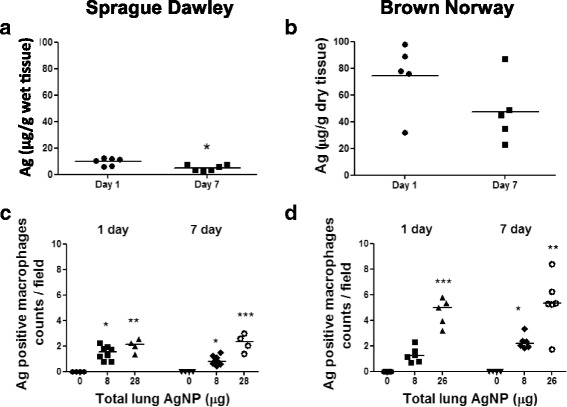
Panels **a** & **b**: Silver quantification using ICP-MS in lung tissue following inhalation of the high dose of freshly-generated silver nanoparticles. In both rat strains, there was a reduction in lung silver content at Day 7 compared to Day 1, although this only reached statistical significance in the Sprague–Dawley rats. The silver lung content was higher in Brown-Norway rats compared to Sprague–Dawley rats. Panels **c** & **d**: Counts of macrophages staining positive for silver in the lung tissue of Sprague Dawley and Brown Norway rats exposed to silver nanoparticles at 1 and 7 days post inhalation at each lung dose deposition. Data for individual rats are shown with the median for each group denoted as a horizontal bar. *P < 0.05, **P < 0.01, ***P < 0.001 versus the air only control within each time-point

Silver levels in the lungs of SD rats exposed to an estimated deposited dose of 28 μg of AgNP particles were 9.97 ± 2.79 μg/g (wet weight) at 24 h. Assuming a typical rat lung weight of 1 g this suggests significant clearance within the first 24 h, and lower levels of 4.99 ± 2.21 μg/g wet weight at day 7 (*P* < 0.05), indicate further clearance had occurred from the lung by this time (Fig. 2 a). The silver concentration in BN rat lung at 24 h was 74.46 μg/g dry weight). Typically lung dry weight is ~20 % of wet weight so this corresponds to around 15ug/g wet weight, which is higher by 50 % compared to that found in the SD rats. For the BN rats, there was a non-significant reduction measured at Day 7 (Fig. 2 b), which suggests slower clearance from the BN rat lung.

In BN rats, there was a clear dose-dependent increase in the number of silver-positive staining cells in the lung at both 1 and 7 days post inhalation, this dose-dependent trend was less clear for the SD rats, particularly at 1 day where the number for both doses were very similar (Fig. 2 c & d). The numbers of silver positive stained cells were between 2 and 3 times higher for the BN compared to the SD rats for the high dose exposure. For both SD and BN rats numbers of stained cells at 1 and 7 days were similar, indicating no consistent time-dependence.

**Fig. 3:**
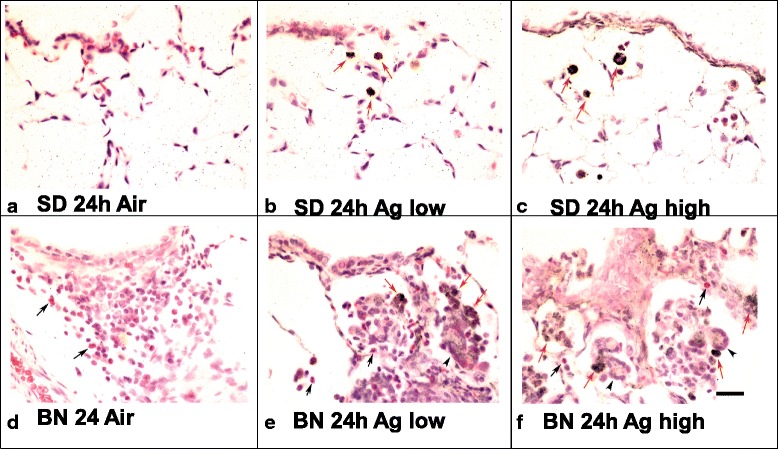
Silver-enhancing and hematoxylin-eosin-stained Sprague–Dawley (SD) and Brown-Norway (BN) rat lung sections at 24 h post-treatment. Panels **a & d**: SD and BN air control shows an absence of signal; silver-stained positive cells are seen as black or black brown positivity (red arrows). In Panel **b**, SD at lung dose of 8 μg; in Panel **c**, at lung dose of 28 μg AgNPs and in Panel **e**, BN at lung dose 8 μg and Panel **f**, 26 μg AgNPs. Positive silver-stained cells are deposited in granuloma of BN lungs (arrow head pointing multi-nuclear giant cells). There are high levels of eosinophilic inflammation (black arrows) in BN lungs (internal scale bar = 20 μm for all)

Silver staining was not observed in non-exposed SD and BN lungs (Fig. 3 a & d). There were black silver particles observed at 24 h post-AgNP inhalation. Silver positivity at 24 h was as strong as at 7 days after AgNPs inhalation in both rat strains. Silver-positive cells consisted mainly of macrophages scattered in the alveolar space and lung interstitium of SD lungs (Fig. 3 b & c). Silver-positive macrophages were embedded within the inflammatory cells which infiltrate the alveolar septa and lamina propria of blood vessels and airway mucosa and were deposited in granulomas of BN lungs (Fig. 3 e & f). Silver particles were also observed on the luminal surface and on surface epithelium and subepithelial connective tissue of terminal bronchioles, scarcely at day 1 but more prominent at day 7 (Fig. 3).

#### Lung inflammatory changes

**Fig. 4:**
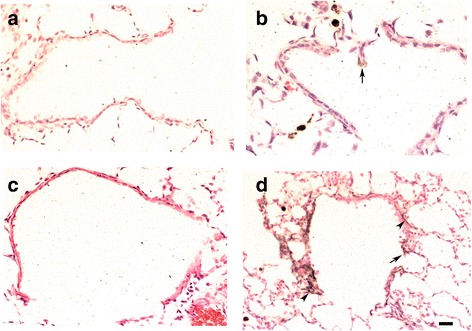
Silver-enhancing and hematoxylin-eosin-stained terminal bronchioles of Sprague–Dawley rat lungs showing no signals in air control at 24 h and 7 days (Panels **a & c**). In Panel **b**, a few black silver particles are deposited on the surface of epithelial cells (arrow) at 24 h after 28 μg AgNPs inhalation. In Panel **d**, more visible agglomerated black silver positivity were observed on the luminal surface and on surface epithelial cells (arrows) and subepithelial connective tissue (arrow heads) at 7 days post-8 μg AgNPs inhalation (internal scale bar = 20 μm for all)

**Fig. 5:**
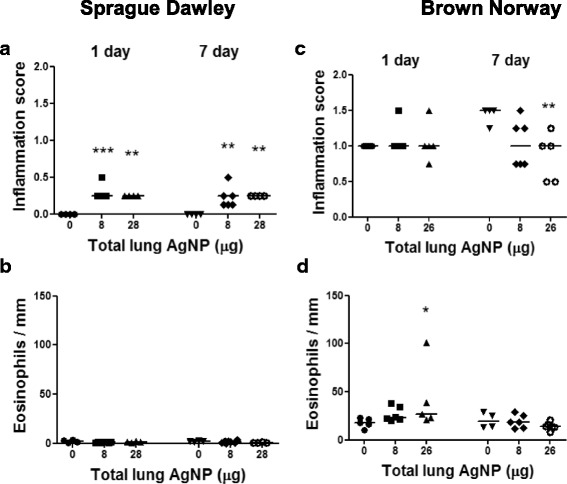
Lung tissue inflammatory scores (Panels **a & b**) and eosinophil counts per mm length of airway wall (Panels **c & d**) in Sprague Dawley and Brown Norway rats exposed to silver nanoparticles at 1 and 7 days post inhalation as a function of lung deposited doses of silver. Individual data-points shown for 8–12 rats per group with horizontal bar showing mean. *P < 0.05, **P < 0.01, ***P < 0.001 versus the air only control (0) within each time-point

Following inhalation of silver, there was a mild inflammation with a few inflammatory cell infiltrations in the bronchial and vascular walls and alveolar septa in SD rat lungs (Fig. 4b & d) compared to the control air-exposed rats (Fig. 4a & c). Overall, tissue inflammation scores were increased in lungs of SD rats at both day 1 and 7 and at both exposure doses with the scores remaining unchanged during that period (Fig. 5a). In BN rats, there was a high level of baseline inflammation that remained unchanged after inhalation of AgNPs at both levels of exposure and on both day 1 and day 7 (Fig. 5c). Areas of inflammation consisting of eosinophils, neutrophils, and mononuclear cells in BN rat lungs could be seen (Fig. 3e & f). Eosinophil counts in the airway wall were increased in BN rats at day 1 after the 28 μg exposure dose but not at day 7 (Fig. 5d). There was no eosinophil increase in the SD lung tissue (Fig. 5b).

#### BAL inflammatory cells

**Fig. 6:**
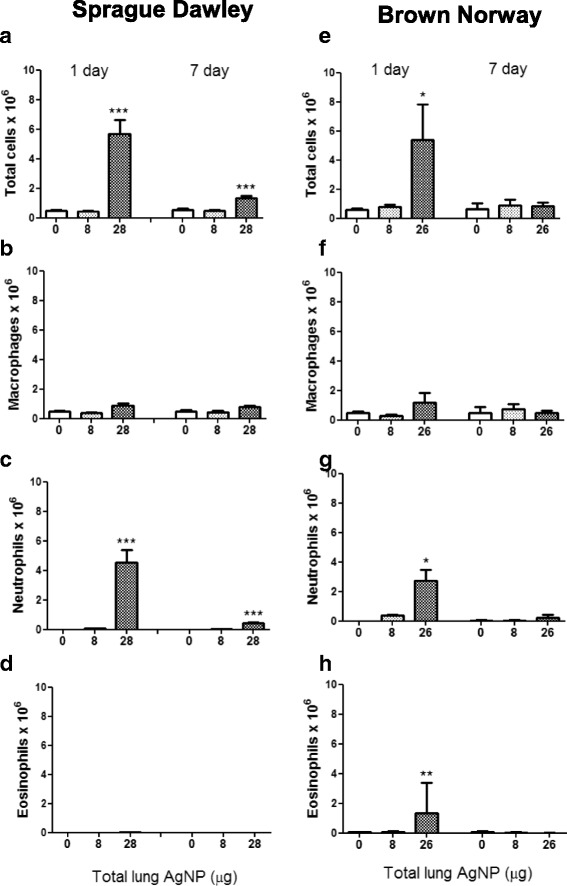
Differential cell counts in bronchoalveolar lavage fluid after inhalation of silver nanoparticles at 1 and 7 days post-inhalation in Sprague Dawley rats (Panels **a-d**) and in Brown Norway rats (Panels **e-h**) as a function of lung dose. Data shown as mean ± SD (*n* = 8–12 rats per group). *P < 0.05, **P < 0.01, ***P < 0.001 versus the Air only control (0) within each time-point

In SD and BN rats, total cell numbers increased at 1 day post inhalation of the high 28 μg AgNP (*P* = 0.0002 & *p* < 0.02, respectively), and in SD rats, this remained elevated at day 7 (Fig. 6). Low doses of AgNP (8 μg) did not elicit an increase in cells. The increase in total cells was reflected in an increase in neutrophils (*P* = 0.0002) in SD rats, while there was both an increase in neutrophils (*P* < 0.02) and eosinophils (*P* < 0.01) in BN rats. Neutrophil numbers fell but remained elevated in SD rats at day 7 (*P* = 0.009). There was also a small increase in lymphocyte numbers at the high dose exposure in SD rats (*P* < 0.03; data not shown).

#### BAL total protein, malonaldehyde (MDA), phospholipid and surfactant proteins

**Fig. 7:**
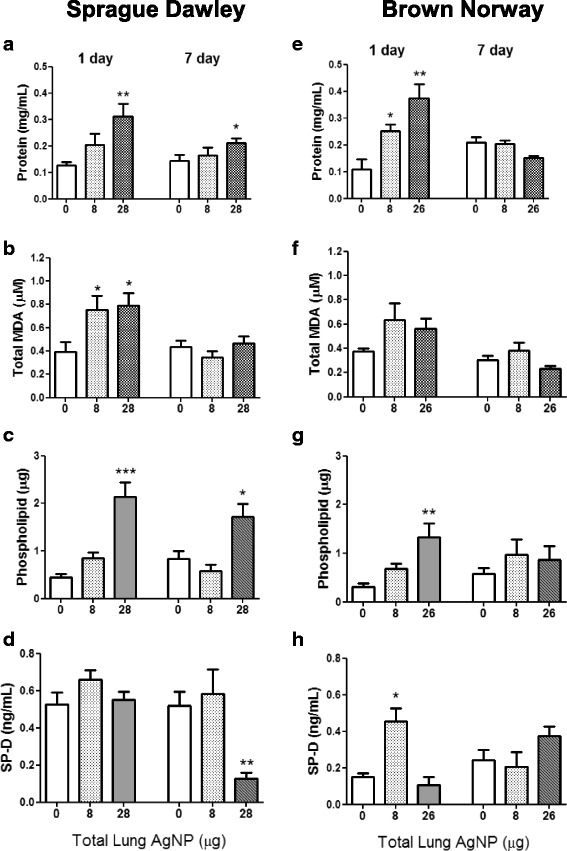
Levels of total protein (Panels **a & e**), malonaldehyde (MDA; Panels **b & f**) and surfactant protein D (SP-D; Panels **d & h**) in bronchoalveolar lavage fluid in Sprague Dawley and Brown Norway rats as a function of deposited lung dose of silver nanoparticles. Phospholipid levels (Panels **c & g**) were measured in the large aggregate fraction. Data shown as mean ± SD (*n* = 8–12 rats per group). *P < 0.05, **P < 0.01, ***P < 0.001 versus the Air only control (0) within each time-point

Total BAL protein increased in the SD rats at 28 μg dose only (*P* < 0.001) at day 1, and in the BN rats at both 8 μg (*P* < 0.02) and 26 μg (*P* < 0.01). Total BAL protein levels returned to baseline levels by 7 daysay in both rat strains (Fig. 7 a & e). In the SD rats, MDA increased at 1 day after inhalation of 8 (*P* < 0.03) and 28 μg (*P* < 0.01) of AgNP, with levels returning to baseline by day 7. There was a similar trend in the BN rats but the increases were not significant (Fig. 7 b & f).

In the SD rat, SP-D levels in whole BAL did not change at 1 day while at 7 days, there was a decrease after the high dose. In the BN rat, SP-D levels increased after the low dose but not after the high dose at 1 day (Fig. 7 d & h). There was no change in SP-D at the 26 μg dose at 7 days for the BN rats. Total phospholipid levels in the large aggregate fraction of BAL increased according to the dose of AgNPs exposed for both rat strains at day 1. By 7 day, phospholipid level remained elevated in the SD rat after the 26 μg dose, while in the BN rat, levels had returned to baseline (Fig. 7 c & g).

#### BAL cytokines/chemokines

**Fig. 8:**
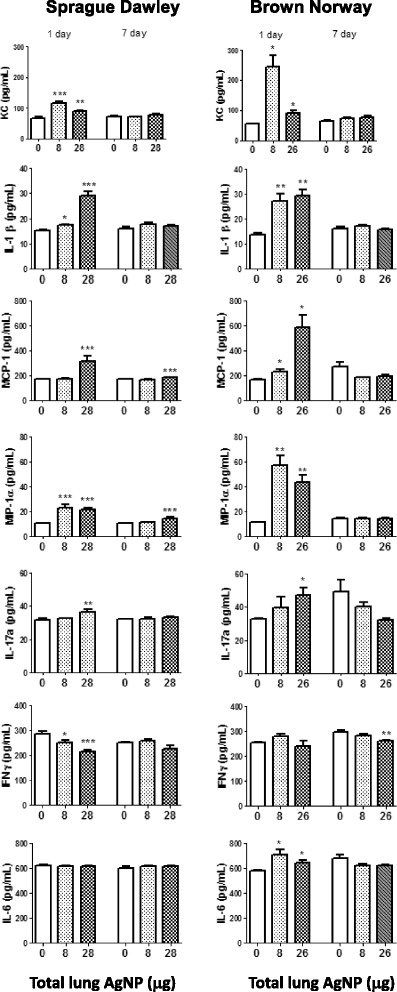
Cytokine levels in bronchoalveolar lavage fluid in Sprague Dawley and Brown Norway rats as a function of deposited lung dose of silver nanoparticles. Data shown as mean ± SD (*n* = 8–12 rats per group). *P < 0.05, **P < 0.01, ***P < 0.001 versus the air only control (0) within each time-point

Most of the changes in cytokine levels occurred at day 1. In the SD rat, levels of IL-1β, KC, IL-17A, CCL2 (MCP-1) and CCL3 (MIP-1α) increased at the 28 μg dose, with increases also seen at 8 μg for IL1β, KC and CCL3 *(*Fig. 8*)*. On the other hand, levels of IFNγ fell in a dose-dependent manner. CCL2 and CCL3 remained elevated at the high dose at day 7. In the BN rat, there were similar trends apart from an additional increase in IL-6 at day 1 at both 8 and 26 μg doses. Levels of KC, CCL2 and CCL3 were generally higher in BN rat compared to SD rat. There were no changes in levels of IL-4, IL-13 and CCL-11 (eotaxin) (*data not shown*).

#### Lung function

**Fig. 9:**
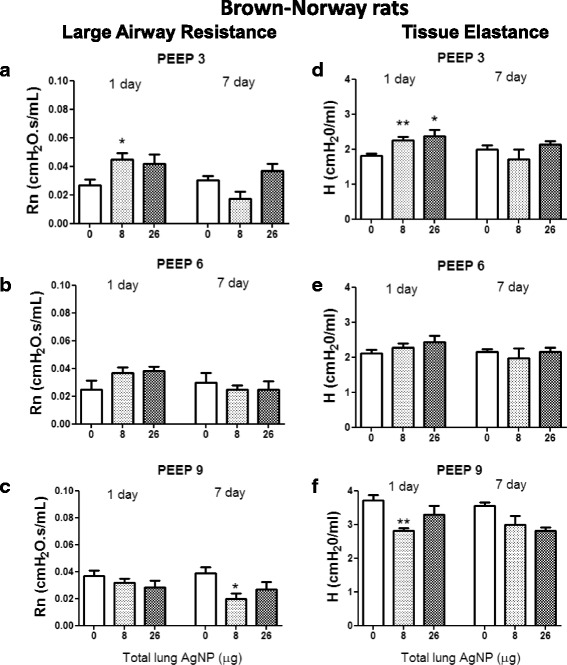
Large airway resistance (Rn) (Panels **a, b & c** *)* and tissue elastance (**h**) (Panels **d, e & f** *)* in Brown Norway rats exposed to silver nanoparticles at PEEP 3, 6 and 9 cm H_2_O, at 1 and 7 days post inhalation as a function of lung deposited doses of silver. Data shown as mean ± SD, *n* = 6 rats per group. *P < 0.05 and **P < 0.01 versus the air only control (0) within each time-point

There were no changes in large airway resistance (Rn), tissue damping (G) or tissue elastance (H) in the SD rats at either time-point compared with the air control at a physiological PEEP of 3 cm H_2_O, suggesting normal airway and parenchymal functioning of the SD lung (*data not shown*). In BN rats, at PEEP 3, Rn increased at both AgNP doses, although this was only statistically significant for the 8 μg dose (Fig. 9 a). Increasing the PEEP from 3 to 6 and 9 cm H_2_O resolved the increase in Rn recorded at PEEP 3 (Fig. 9 b & c). On the other hand, a small non-significant decrease in Rn at 7 days at PEEP 3 for the 8 μg dose, was not resolved by PEEP suggesting some ongoing effects on lung function. There was also a significant increase in H for BN rats at PEEP 3 at the highest dose (Fig. 9 d), suggesting effects on mechanical pulmonary function also originated in the parenchyma and that the BN rats had stiffer lungs. Again, increasing the PEEP from 3 to 6 and 9 cm H_2_O, resolved the increases in H, suggesting that this may be due to a recruitment phenomenon as before (Fig. 9 e & f). Similar to the SD rats, there were no changes in G in the BN rats suggesting that no parenchymal distortion had occurred (*data not shown*).

## Discussion

In this study, we report that inhalation of freshly-generated silver nanoparticles of spherical shape of 15 nm diameter induced an acute pulmonary neutrophilic inflammation with the production of proinflammatory and pro-neutrophilic cytokines in SD and BN rats. However, there were differences between the 2 strains in that the BN rat also showed an eosinophilic inflammation and also was the only strain to respond with a deterioration of lung function. In addition, our study further delineates the differential response of the lungs of these 2 rat strains to inhalation of silver nanospheres in relation to the clearance of silver from the lungs and the phospholipid and surfactant production.

### Silver tracing and clearance in the lungs

The number of cells, particularly macrophages, containing silver increased with dose exposure in both strains, with the number of silver-positive cells in the BN rat 2 to 3-fold higher than in the SD rat at each time-point for the high dose. The uptake of nanoparticles into macrophages is likely to result in oxidative stress, the release of pro-inflammatorymediators and subsequent pulmonary inflammation [15, 29, 30]. Concomitantly, we found that the amount of silver in the lungs persisted to the seventh day, despite a small reduction by that time, but the levels found in the lungs of Brown-Norway rats were approximately 50 % higher than in the lungs of Sprague–Dawley rats after the high dose exposure. The levels of silver measured in our Sprague–Dawley rats were higher than those previously reported in Sprague–Dawley or Fischer rats exposed to spark-generated uncoated silver nanoparticles of a similar size, partly due to the higher levels of exposure we delivered in our study [2, 6, 7, 31, 32]. The calculation of silver levels from the day 1 and day 7 measurements in the Sprague–Dawley rats indicate a clearance of 50 %, which is in a similar range to the clearance of 15 nm spark-produced AgNPs inhaled by Fischer rats at a lower concentration of 179 μg/m^3^ (compared to ~700 μg/m^3^ for our study) for a similar exposure pattern (6 h/day for 4 days) of 62 % between 1 and 7 days post exposure [6]. Clearance of such uncoated AgNPs have been reported to be much faster, for example in the study of Takenaka et al. [2], it was 62 % one day after exposure, increasing to 96 % on day 7 (clearance between 1 day and 7 days was 89 %) in Fischer rats. In a recent study of nebulised AgNPs of primary particle size 20 nm suspended in citrate buffer with a high mass concentration of 7.2 mg/m^3^ delivered to Sprague–Dawley rats for 6 h, a clearance of 34 % between day 1 and day 7 was reported [33]. The comparison between different studies is difficult because of differences in amount of exposure, dose of nanoparticles, method of generation of nanoparticle aerosols, particle size and even animal gender, but most of these studies would indicate that the silver persists for a period of at least 7 days after inhalation. In the study of Anderson et al. [33], one third of the initial silver inhaled persisted even at 56 days, and this was associated with a greater persistence of silver-positive macrophages at 21 and 56 days. In our study, the clearance between 1 day and 7 days for the Brown-Norway rat compared to the Sprague–Dawley rat was lower at 33 %, and was associated with a 2 to 3-fold higher number of silver-containing lung macrophages compared to SD lungs. It is possible that the pre-inflamed lung of the Brown-Norway rat may retain more nanoparticles with a slower clearance rate leading to higher levels of silver found in the lungs when compared to the Sprague–Dawley rat.

### Phospholipid and surfactant levels and influence on lung function

In accord with previous reports of the inhalation of nanoparticles of titanium oxide, silica or cadmium oxide or ultrafine diesel exhaust particles in rats or mice [34–37], we found that inhalation of AgNPs dose-dependently induced an increase in the levels of phospholipids and total protein in bronchoalveolar lavage fluid maximal at day 1 to a similar extent in both strains. Coating of the AgNPs with phospholipid may have important implications on pulmonary homeostasis by interfering with the biophysical surfactant function such as decreased adsorption of pulmonary surfactant at the air-liquid interface as has been observed for gold nanoparticles [23]. On the other hand, we found that the inhalation of AgNPs resulted in diametrically-different effects on the measured levels of SP-D in BAL fluid. While there was a decrease in SP-D levels in Sprague–Dawley rats at day 7 following the high dose exposure, there was an increase in SP-D levels in BAL from Brown-Norway rats at the low dose exposure at day 1, with a non-significant increase at the high dose at day 7. Interestingly, the levels of SP-D at baseline in BAL fluid was nearly 3-fold higher in Sprague–Dawley rats compared to Brown-Norway rats. The reduction in SP-D in Brown-Norway rats may be due to increased SP-D turnover by alveolar macrophages, possibly as a result of binding to AgNPs [22]. The acute increase in SP-D levels in day 1 seen in Brown-Norway rat may represent an increased production from alveolar Type II cells. Increased amounts of SP-D may lead to increased aggregation of nanoparticles [21], hence targeting them towards macrophage and lung clearance [38]. In a recent study, we have also shown that incubating AgNPs with Curosurf^R^ reduced the amount of IL-6 and IL-8 release from human alveolar Type 1 cells exposed to these AgNPs [39].

Lung function changes only occurred in Brown-Norway rats indicating a greater sensitivity of the airways and lungs to the effect of inhaled AgNPs, similar to the lung function responses observed with instillation of AgNPs [15]. Resolution of the increase in lung resistance recorded at low positive end-expiratory pressure (PEEP) by increasing PEEP suggests that this increase was a recruitment phenomenon which affected dynamic breathing that may be related to surfactant dysfunction rather than inherent forces in the lung which can occur following lung injury [40].

### Inhalation versus direct instillation

The results of inhalation of AgNPs in the present study can be compared with our own work on the effect of direct instillation of these nanoparticles into the lungs [15]. In the previous study, we instilled AgNPs at 20 nm diameter capped with citrate or polyvinylpyrrolidone (PVP), the diameter nearest to the spark-generated particles at an intra-tracheal dose of 30 μg in both strains. Indeed, the pattern of responses in these 2 strains in terms of the inflammatory response and of lung function changes were similar for both strains, albeit a lesser response by the inhaled route when a maximal dose of 26–28 μg was deposited in the lungs. The highest inhaled dose used at which we saw an effect was acquired over a period of 12 h of inhalation, while for the instillation, a larger bolus dose was administered over seconds. Thus, with the instilled dose of both citrate and pvp-capped 20 nm silver nanoparticles, we saw a neutrophilic response at day 1 in both strains but only eosinophilic response significant at day 7 in Sprague–Dawley rats. Similarly, there was an increase in protein concentration and levels of MDA in bronchoalveolar lavage fluid, with increased levels of KC at day 1 in both strains. Changes in lung function were only seen in Brown-Norway rats at day 1 also, but not in Sprague–Dawley rats, changes that were similar as those observed in the current study. The inflammatory data we have observed are simialr to those reported from a study of Silva et al. [41] who reported that instillation of AgNPs into the lungs of rats induced an inflammatory response at day 7 with resolution by day 21, while at day 56, there was no inflammatory response and no evidence of airway wall remodelling. By contrast, Song et al. [10] reported that in Sprague–Dawley rats exposed to spark-generated AgNP aerosol, there was persistence of mild inflammation observed at 12 weeks. Longer term exposure studies are needed.

## Conclusions

The pre-existing inflammatory state of the Brown-Norway rat is likely to underlie the increased amount of silver retained in the lungs with a reduced clearance rate that may underlie the increased inflammatory response, induction of SP-D and phospholipid and airway and parenchymal dysfunction observed in this rat strain but not in Sprague–Dawley rats. Our findings would indicate that inhalation of AgNPs in people with pre-existing inflammation in the lungs such as asthma patients or those with chronic obstructive pulmonary disease would lead to a greater degree of inflammation with heightened consequences on lung function.
